# Incidental Asymptomatic Splenic Infarct in a COVID-19 Patient

**DOI:** 10.7759/cureus.13065

**Published:** 2021-02-01

**Authors:** Natasha Ghalib, Prateek Pophali, Natalia Chamorro-Pareja, Apoorva Jayarangaiah, Abhishek Kumar

**Affiliations:** 1 Internal Medicine, Albert Einstein College of Medicine/Jacobi Medical Center, Bronx, USA; 2 Hematology/Oncology, Albert Einstein College of Medicine/Jacobi Medical Center, Bronx, USA

**Keywords:** covid-19, splenic infarct, arterial thrombosis

## Abstract

A high incidence of thromboembolic events and coagulation parameter abnormalities are seen in cases of coronavirus disease 2019 (COVID-19). Both venous and arterial thrombosis, including arterial thrombosis in unusual sites, have been reported in COVID patients in recent literature. Herein, we report a case of a 67-year-old female patient with non-critical COVID-19 disease with an incidental finding of an asymptomatic splenic infarct. In the absence of a cardio-embolic source, we believe this was an arterial thromboembolic event in the splenic circulation. The duration and modality of anticoagulation of inpatient and ambulatory COVID patients remains a dynamic discussion. Our case adds the evidence of a clinically silent arterial thrombotic event in a non-critical COVID-19 patient which further emphasizes the need to address the strategies for diagnosis and management of thrombo-embolism to prevent potentially fatal complications.

## Introduction

The United States, amongst other countries, has been severely affected by the coronavirus disease 2019 (COVID-19) pandemic caused by the severe acute respiratory syndrome coronavirus 2 (SARS-CoV-2). COVID-19 is primarily a respiratory disease with multisystem involvement and a high mortality rate, especially amongst hospitalized patients (21%) [1]. Patients with COVID-19 infection harbor a hypercoagulable state as part of the disease spectrum [2]. This hypercoagulable state is due to the systemic inflammation induced by the elevated cytokines causing endothelial activation [3]. Another pathogenic mechanism for the pro-thrombotic state is activation of hypoxia-inducible transcription factors (HIFs) [4]. As we learn more about the biology of COVID-19, more pathogenic mechanisms for the procoagulant state are being reported. There are multiple reports of clinically significant thrombosis in critically ill patients [2,5-7]. Herein, we report a case of a non-critical COVID-19 patient admitted to a New York City public hospital with an incidental finding of splenic infarction.

## Case presentation

A 67-year-old female with a past medical history of hypertension, diabetes mellitus, coronary artery disease, hypothyroidism, and mild intermittent asthma was admitted to our hospital with a one-week history of progressively worsening shortness of breath, hypoxia, and fever. She also noted pain in her left calf for a similar duration without any erythema or edema. She denied recent hospitalization, surgery, trauma, or a history of clots. She had exposure from her mother, who recently passed away due to COVID-19. Her vitals on presentation were temperature 101F, heart rate 100 beats/min, blood pressure 127/76 mmHg, respiratory rate 45/min, and oxygen saturation 64% which improved to 97% on the non-rebreather mask. Her physical examination was only significant for pain on palpation of the left calf with erythema, edema, and normal distal pulses.

Initial investigations revealed a complete blood count with hemoglobin 11.4 gm/dl, white blood cell count 10.8/nL (80% neutrophils, 10% lymphocytes), platelet count 465/nL, prothrombin time (PT) 13.9 sec, international normalized ratio (INR) 1.2, partial thromboplastin time (PTT) 19.1 sec and D-dimer 1072 ng/mL, ferritin 536 ug/L, C-reactive protein (CRP) 163.3 mg/L. COVID-19 testing with reverse transcription polymerase chain reaction (RT-PCR) was positive. Chest X-ray showed bilateral ill-defined hazy infiltrates characteristic of COVID-19.

A computed tomography (CT) angiogram of the chest and lower extremity venous duplex was obtained due to high clinical suspicion for pulmonary embolism (Wells’ score: 6, moderate risk group) and deep venous thrombosis (DVT) (Wells’ score: 1, moderate risk group), respectively. Both the studies showed no evidence of a pulmonary embolus or DVT, however, CT angiogram of the chest captured a wedge-shaped area of low attenuation in the medial aspect of a normal-sized spleen, suggestive of a splenic infarct.

**Figure 1:**
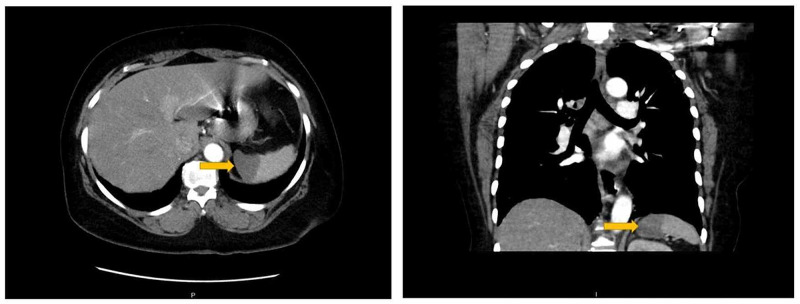
Axial and coronal view of the CT chest-abdomen showing area of low attenuation on the medial side of the spleen

A comparison to imaging from less than a month ago revealed a normal spleen on an ultrasound of the abdomen. Throughout her hospitalization, she denied abdominal pain, nausea or vomiting, and her abdominal examination remained benign.

The patient did not have any evidence suggestive of a cardio-embolic etiology for the splenic infarct. She did not have evidence of atrial fibrillation nor any stigmata of infectious endocarditis. Transthoracic echocardiogram did not show evidence of valvular lesions, right to left shunts or an intramural thrombus. Evaluation for anti-phospholipid syndrome showed a negative lupus anticoagulant, low positive cardiolipin immunoglobulin G (IgG) antibody (24 U/ml), and a positive beta-2 glycoprotein 1 immunoglobulin M (IgM) antibody (25.6 SMU).

The patient was administered therapeutic anticoagulation with continuous heparin infusion. After starting anticoagulation, D-dimer decreased to 427 ng/mL. After symptomatic management and resolution of hypoxia over seven days, the patient was discharged home on therapeutic anticoagulation with low molecular weight heparin (enoxaparin) with outpatient hematology follow up.

## Discussion

COVID-19 is known to be a hypercoagulable state. Abnormal coagulation parameters, including elevated D-dimer, fibrinogen degradation products (FDP), low Factor XII levels, and positive lupus anticoagulant are well described in the literature to be associated with the risk for thrombosis [7-9]. Elevated D-dimer and FDP levels are associated with a poor prognosis [7,9]. There is a high incidence of venous thromboembolism, with most studies reporting an incidence between 20-27% [2,5-6]. However, a French study reported rates as high as 69% in COVID-19 patients needing intensive care unit (ICU) level of care using a complete duplex ultrasound [10]. An Italian study reported a cumulative venous/arterial thromboembolic event rate of 6.6% in 314 patients admitted to the general medicine wards [6]. Amongst these, arterial thromboembolic events included stroke (1.9%) and acute coronary syndrome (1%) [6]. Another study on ICU patients in the Netherlands reported arterial thrombosis rate to be 3.7% (all ischemic strokes) [5]. Except for these studies, data on the incidence of arterial thromboembolic events, especially in the splanchnic and peripheral vasculature, is scarce.

The presence of cardiolipin antibody and beta-2 glycoprotein 1 antibody could be a transient phenomenon in the setting of an acute infection. The elevated CRP does not predict the transient or persistent nature of these antibodies as shown by Twito et al. [11]. The patient needs re-testing after 12 weeks to establish the diagnosis of antiphospholipid syndrome. Zhang et al. recently described three cases with a negative lupus anticoagulant and positive cardiolipin and beta-2 glycoprotein 1 antibody in ICU patients with significant thrombotic disease, however, antibody titers were not mentioned in their report [12]. Since low titers are rarely associated with thromboembolism and are not considered in the Sapporo criteria [13], the relation of cardiolipin and beta 2 glycoprotein 1 antibodies with thrombo-embolic events in COVID-19 also needs further elucidation as we are dealing with a novel disease with potentially unknown pathological mechanisms. Endothelial inflammation from direct infection via viral entry through angiotensin-converting enzyme 2 (ACE-2) receptors present on endothelial cells has also been implicated in arterial thrombosis in COVID-19 patients [14].

In our case, the presence of a splenic infarct in the absence of a cardio-embolic source points to a primary arterial thromboembolic event in the splenic circulation due to COVID-19 related hypercoagulability. It adds to the evidence of a clinically silent arterial thrombotic event in a non-critical COVID-19 patient which further emphasizes the need to re-address the strategies for diagnosis and management of thrombo-embolism to prevent potentially fatal complications [15].

## Conclusions

COVID-19 infection predisposes a hypercoagulable state with high incidence of both venous and arterial thrombosis. Splenic infarcts associated with COVID-19 have been rarely reported. A degree high of suspicion must be maintained in patients presenting with acute abdominal pain.
